# Influence of Ligand Environment Stoichiometry on NIR-Luminescence Efficiency of Sm^3+^, Pr^3+^ and Nd^3+^ Ions Coordination Compounds

**DOI:** 10.3390/molecules28155892

**Published:** 2023-08-05

**Authors:** Trofim Polikovskiy, Vladislav Korshunov, Mikhail Metlin, Viktoria Gontcharenko, Darya Metlina, Nikolay Datskevich, Mikhail Kiskin, Yury Belousov, Alisia Tsorieva, Ilya Taydakov

**Affiliations:** 1 P. N. Lebedev Physical Institute of the Russian Academy of Sciences, 53 Leninskiy 1. Prospect, 119991 Moscow, Russia; polikovskiy.ta@gmail.com (T.P.); mikhail.metlin@gmail.com (M.M.); victo.goncharenko@gmail.com (V.G.); fekla.andreevna@gmail.com (D.M.); dac1@yandex.ru (N.D.); or belousov@inorg.chem.msu.ru (Y.B.); tsorievaav@gmail.com (A.T.); taidakov@mail.ru (I.T.); 2 Faculty of Chemistry, National Research University Higher School of Economics, 20 Miasnitskaya Str., 101000 Moscow, Russia; 3 Kurnakov Institute of General and Inorganic Chemistry, Russian Academy of Sciences, 119991 Moscow, Russia; m_kiskin@mail.ru; 4 Chemistry Department, M.V. Lomonosov Moscow State University, Leninskie Gory Str., Building 1/3, 119991 Moscow, Russia

**Keywords:** Lanthanides, coordination compounds, luminescence, Judd–Ofelt theory

## Abstract

Six new complexes of the ligand HQ^cy^ (-4-(cyclohexanecarbonyl)-5-methyl-2-phenyl-2,4-dihydro-3H-pyrazol-3-one) and Ln^3+^ ions with emission in the near-infrared (Nd^3+^) or visible and near-infrared (Sm^3+^, Pr^3+^) spectral regions were synthesized and characterized using various methods, including single crystal X-ray diffraction. The study demonstrated that both tris complexes [LnQ^cy^_3_(H_2_O)(EtOH)] and tetrakis-acids [H_3_O][LnQ^cy^_4_] can be synthesized by varying the synthetic conditions. The photochemical properties of the complexes were investigated experimentally and theoretically using various molecular spectroscopy techniques and Judd–Ofelt theory. The objective was to quantitatively and qualitatively disclose the influence of complex stoichiometry on its luminescence properties. The study showed that the addition of an extra ligand molecule (in the tetrakis species) increased molar extinction by up to 2 times, affected the shape of photoluminescence spectra, especially of the Pr^3+^ complex, and increased the quantum yield of the Sm^3+^ complex by up to 2 times. The results obtained from this study provide insights into the luminescent properties of lanthanide coordination compounds, which are crucial for the design and development of novel photonic materials with tailored photophysical properties.

## 1. Introduction

Nowadays, there is a high demand for creating new highly efficient sources of emission at the near-infrared (NIR) [1,2] or both the NIR and visible spectral regions [3,4]. One of the prominent material classes for such purposes is lanthanide coordination compounds with organic ligands. Such interest is related to optoelectronic [5,6,7,8], spectroscopic and photonic applications [9,10,11] due to the relatively high luminescence efficiency and narrow emission bands typical to trivalent lanthanide ions [9,10,11]. However, lanthanide ion’s luminescence intensity is limited by Laporte selection rules [12]. Coordinating lanthanide ions with organic molecules usually significantly increases the luminescence efficiency of the ions. This happens due to the “antenna effect” [13], which is based on the electronic excitation energy transfer from a ligand to an ion, partially allowing the forbidden f ⟶ f* transition. Interestingly, intensities for the f ⟶ f* transitions in compounds with a high-symmetry coordination polyhedron tend to be weaker than those with low symmetry [14].

Acylpyrazolones have previously been studied as chelating ligands, heterocyclic analogues of β-diketones, effectively sensitizing the luminescence of terbium [15,16,17] and dysprosium [3,18,19] ions. This is possible due to the relatively high triplet state energy of the ligands [20,21] compared to conventional β-diketones such as dibenzoylmethane and tenoyltrifluoroacetone [22,23]. In addition, there are separate works devoted to the emission properties of complexes of samarium [20,24] and neodymium [24,25], but this area remains poorly investigated. The luminescence of acylpyrazolonates of praseodymium has not been studied before. Moreover, there are few systematic studies of the photophysical properties of coordination compounds based on Pr^3+^ ions [3,26].

Regarding chemical structure, acylpyrazolones form complexes of different composition: neutral tris-complexes [LnQ_3_(solv)_1–2_] [21,24,25,27,28,29,30,31], adducts with dipyridyl, phenanthroline or phosphinoxides [LnQ_3_(L)], binuclear [Ln_2_Q_6_] [32,33], obtained in the absence of coordinating solvents and M[LnQ_4_] tetrakis-acid anions, where M is an hydroxonium [3,20,34,35], alkylammonium [25,36] or silver cation [20]. Easily formed tris-complexes, in which the coordination number of lanthanide takes values of 7 or 8 depending on steric hindrance of the ligand, were investigated in the best way [37]. Unfortunately, tris-complexes contain coordinated solvent molecules (water or alcohols), so their luminescence is attenuated by phonon-beam relaxation processes [3]. Tetrakis compounds are barely described and their photophysical properties have practically not been investigated in the literature [3,20,38,39]. Constructing a tetrakis compound, we modify luminescence properties of compounds by three factors. Firstly, replacing two water molecules with an additional neutral ligand molecule leads to suppression of the nonradiative multiphonon relaxation on the O-H group oscillations [40]. Secondly, in general, a tetrakis compound is not electrically neutral. Therefore, a cation in a compound may influence the electronic structure of organic ligands. On the other hand, an additional ligand molecule can alter the crystal symmetry of a coordination polyhedron [41,42,43].

**Scheme 1:**
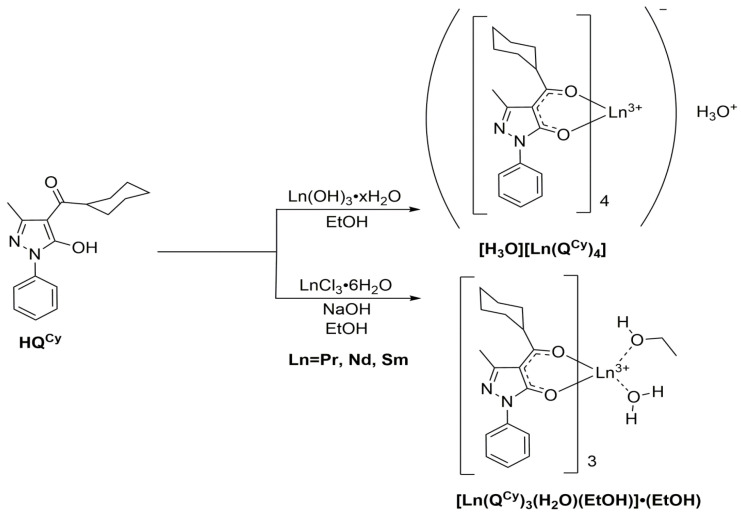
Synthesis of complexes.

In the present work, a series of tris and tetrakis coordination complexes of Sm^3+^, Pr^3+^ and Nd^3+^ ions with methanoncyclohexyl(5-hydroxy-3-methyl-1-phenyl-1H-pyrazo-4-yl)methanon were synthesized (see Scheme 1) and widely investigated using various methods of molecular spectroscopy. The main goal was to quantitatively and qualitatively compare the photophysical parameters of tris and tetrakis coordination compounds pairwise, such as photoluminescence quantum yields, radiative and non-radiative processes rates in dependence on the amount of ligand molecules coordinating each ion.

## 2. Results

### 2.1. Crystal Structure

As all lanthanide complexes are isostructural (Table S1), structure description will only be given for [Sm(Q^cy^)_3_(H_2_O)(EtOH)]∙(EtOH) and [H_3_O][Sm(Q^cy^)_4_].

#### 2.1.1. Tris-Complexes

**Figure 1:**
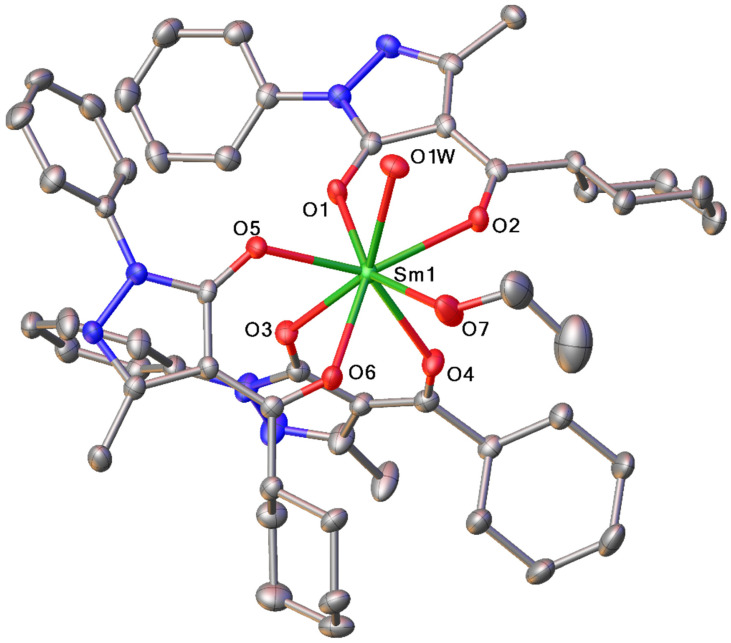
View of the asymmetric unit of [Sm(Q^cy^)_3_(H_2_O)(EtOH)]∙(EtOH). Hydrogen atoms and solvent ethanol molecule are not shown for clarity; thermal ellipsoids are illustrated at 50% probability. Central metal atom and oxygen atoms of ligands are labeled.

**Figure 2:**
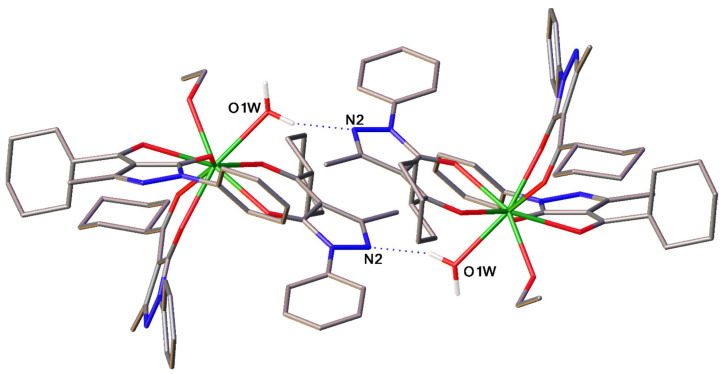
View of the crystal packing of [Sm(Q^cy^)_3_(H_2_O)(EtOH)]∙(EtOH). Hydrogen atoms not involved in intermolecular interactions are not shown for clarity; solvent ethanol molecules as well as thermal ellipsoids are not illustrated. Hydrogen bonds are shown with dotted lines.

The structure is a mononuclear complex (See Figure 1), where the lanthanide ion is coordinated by oxygen atoms of three ligands (O1–O6) and two oxygen atoms of solvate molecules (O7 of ethanol and O1W of water), leading to the octa-coordinated complex with the coordination polyhedron {SmO_8_} which is best described as a square antiprism. Additionally, the structure contains one solvated molecule of ethanol, which is involved in hydrogen bonding with the water molecule (O1W…O1S distance is 2.70 Å). Upon further analysis of the crystal packing, the presence of intermolecular hydrogen bonds between the water molecule and pyrazole fragment of the adjacent molecule of the complex (O1W…N2 distance is 2.83 Å) was revealed, leading to the formation of hydrogen-bonded centrosymmetric dimers (See Figure 2).

#### 2.1.2. Tetrakis-Complexes

**Figure 3:**
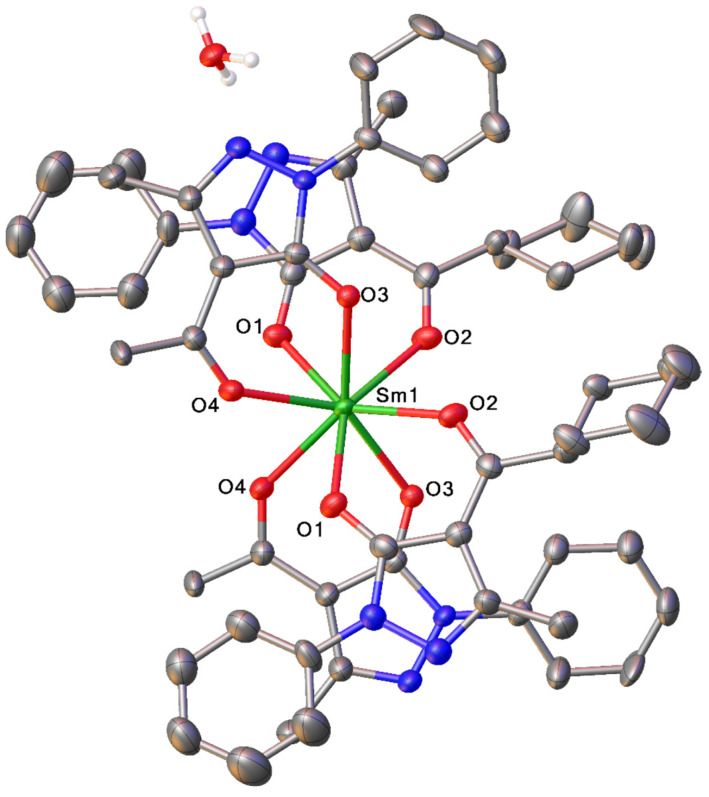
General view of [H_3_O][Sm(Q^cy^)_4_]. Hydrogen atoms at carbon atoms and disordered cyclohexyl fragments are not shown for clarity; thermal ellipsoids are illustrated at 50% probability. Central metal atom and oxygen atoms of ligands are labelled.

**Figure 4:**
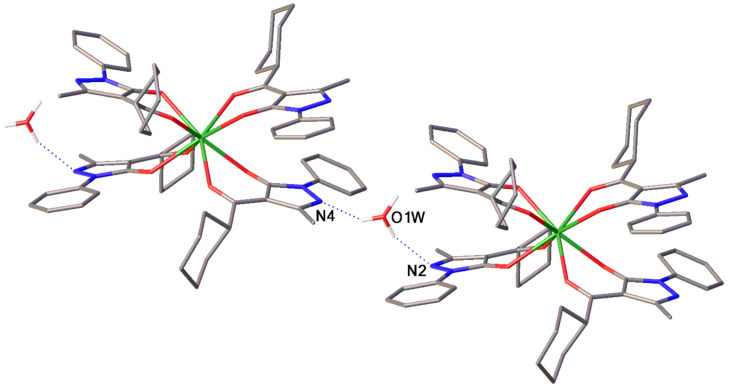
View of the crystal packing of [H_3_O][Sm(Q^cy^)_4_]. Hydrogen atoms not involved in intermolecular interactions are not shown for clarity; disordered cyclohexyl fragments as well as thermal ellipsoids are not illustrated. Hydrogen bonds are shown with dotted lines.

This structure is a mononuclear complex (See Figure 3), where the lanthanide ion is coordinated by oxygen atoms of four ligands, leading to an octa-coordinated negatively charged complex [Sm(Q^cy^)4]^−^, while the hydronium cation (H_3_O)^+^ is present as a counterion. The {SmO_8_} coordination polyhedron is best described as a square antiprism. Additionally, the complex lies on a two-fold rotation axis, and only half of the molecule lies in the asymmetric unit of the crystal structure. Additional proof of the electric negativity of the complex can be found from the bond length analysis of the diketone fragment (Table S2). A rather small difference in C–O and C–C bond lengths indicates that this fragment possesses delocalized negative charge. The analysis of crystal packing revealed that the hydronium cation is involved in two intermolecular hydrogen bonds with nitrogen atoms of pyrazole fragments (O1W…N2 distance is 2.78 Å and O1W…N4 distance is 2.59 Å) of two adjacent molecules of the complex, leading to the linear polymer-like crystal packing (See Figure 4).

### 2.2. Optical Absorption

**Figure 5:**
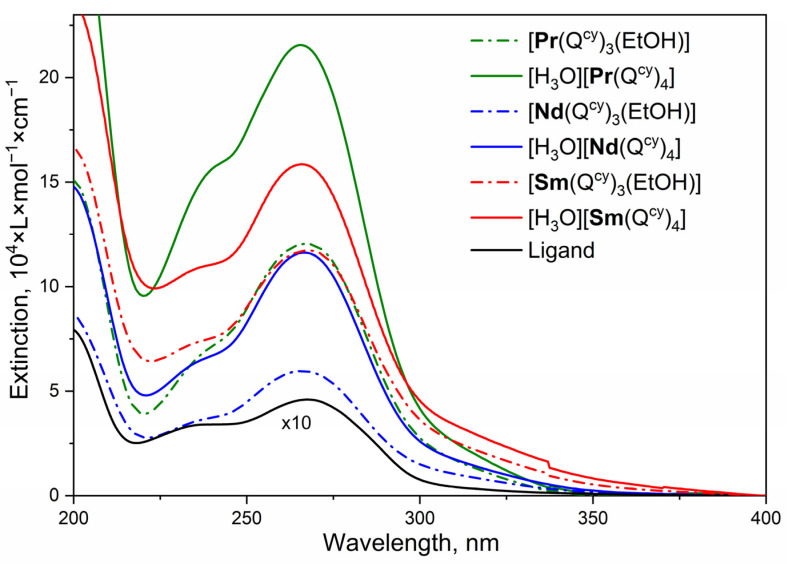
UV–VIS absorption spectra for the compounds in MeCN.

Optical absorption spectra, obtained for HL and complexes [Pr(Q^cy^)_3_(H_2_O)(EtOH)]∙(EtOH), [H_3_O][Pr(Q^cy^)_4_], [Nd(Q^cy^)_3_(H_2_O)(EtOH)]∙(EtOH), [H_3_O][Nd(Q^cy^)_4_], [Sm(Q^cy^)_3_(H_2_O)(EtOH)]∙(EtOH) and [H_3_O][Sm(Q^cy^)_4_] dissolved in MeCN, are shown in Figure 5. All the spectra qualitatively resemble each other. A pronounced maximum is observed at 268 nm. It was found that the coordination of lanthanide ions by this ligand leads to a significant increase in the molar extinction of the ligand environment of 40 times in comparison with the free HL ligand. Energies of the first excited singlet states were estimated as the edge of the low-energy band of the absorption spectra by a well-known tangent method [30]. For this purpose, the spectra were deconvoluted on Gaussian components. The S_1_ energy values for all the compounds are similar and oscillate about 28,500 cm^−1^. The increase in molar extinction is explained by an increase in the oscillator strength value of S_0_ ⟶ S_1_ transition due to the influence of heavy ions on ligand wave functions for the ground and excited states.

Additionally, as the spectra obtained for tris and tetrakis complexes are qualitatively similar, it is clear that the optical absorption of complexes is related to ligand absorption. Notably, adding an additional organic ligand molecule leads to an increase in molar extinction by a factor of two for [Nd(Q^cy^)_3_(H_2_O)(EtOH)]∙(EtOH) and [H_3_O][Nd(Q^cy^)_4_] complexes, 1.4 for [Sm(Q^cy^)_3_(H_2_O)(EtOH)]∙(EtOH) and [H_3_O][Sm(Q^cy^)_4_], and by almost two for [Pr(Q^cy^)_3_(H_2_O)(EtOH)]∙(EtOH) and [H_3_O][Pr(Q^cy^)_4_] complexes.

### 2.3. Photoluminescence

Photoluminescence (PL) spectra for all the compounds were recorded under CW excitation at 340 nm. The spectra reveal numerous narrow emission bands, originated by f ⟶ f* transitions in ions. The interrelation of the following spectral bands with transitions was established according to the literature [44].

**Figure 6:**
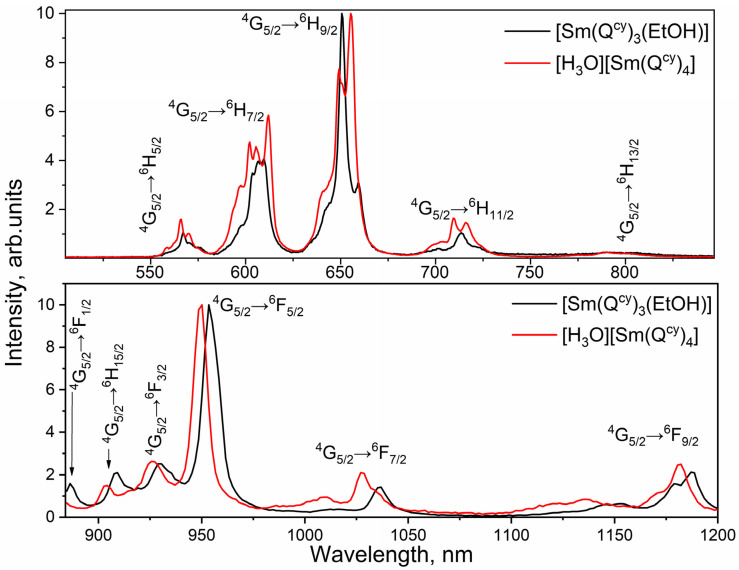
Photoluminescence spectra for the Sm^3+^ ion compounds in solid state under CW excitation at 340 nm.

Photoluminescence (PL) spectra of [Sm(Q^cy^)_3_(H_2_O)(EtOH)]∙(EtOH) and [H_3_O][Sm(Q^cy^)_4_] complexes in the visible and near-IR regions are shown in Figure 6. We observed narrow spectral bands, typical for f ⟶ f* transitions of the Sm^3+^ ion:  G5/2 4→H5/2 6 (555–620 nm), G5/2 4→H7/2 (620–625 nm), 6G5/2 4→H9/2 (625–670 nm), 6G5/2 4→H11/2 (690–730 nm), 6G5/2 4→H13/2 (780–800 nm), 6G5/2 4→F1/2 (880–895 nm), 6G5/2 4→H15/2 (895–920 nm), 6G5/2 4→F3/2 (920–940 nm), 6G5/2 4→F5/2 (940–975 nm), 6G5/2 4→F7/2 (975–1050 nm), 6G5/2 4→F9/2 (1100–1200 nm) 6. There was no ligand fluorescence in the PL spectra of both complexes, which evidences the high efficiency of energy transfer from the ligand to the ion. In addition, the observed emission bands are strongly split into several sub-bands due to the Stark effect [45]. The spectra of [H_3_O][Sm(Q^cy^)_4_] reveal more sub-bands than the spectra of [Sm(Q^cy^)_3_(H_2_O)(EtOH)]∙(EtOH), which may indicate that the tetrakis complex has lower coordination polyhedron symmetry in comparison with the tris complex [20]. However, as calculated by the Shape software (https://shapesoftware.com, accessed on 1 July 2023) [46], the symmetry point group of the coordination polyhedron {MO7} is a square antiprism (D_4d_) in both cases.

**Figure 7:**
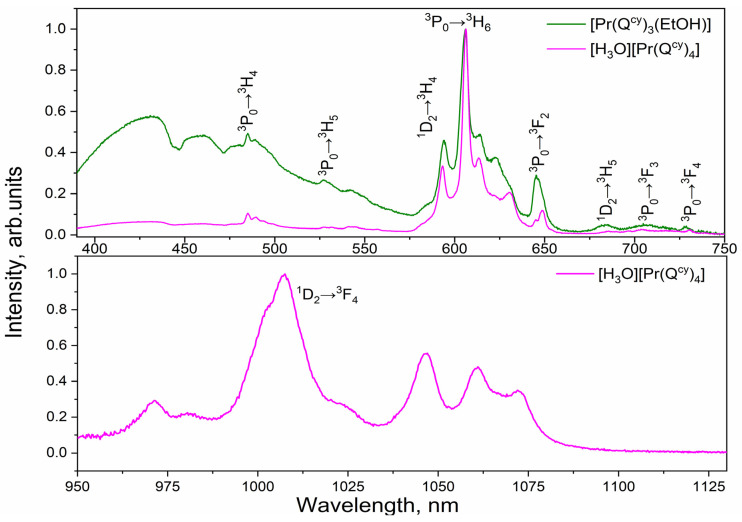
Photoluminescence spectra for the Pr^3+^ ion compounds in solid state under CW excitation at 340 nm.

The PL spectra of the [Pr(Q^cy^)_3_(H_2_O)(EtOH)]∙(EtOH) and [H_3_O][Pr(Q^cy^)_4_] complexes are shown in Figure 7. There are narrow spectral emission bands of the Pr^3+^ ion detected in the visible region of the luminescence spectra of complexes, as well as wide ligand luminescence bands in the blue-green region of the spectrum. The observed narrow emission bands centered at 485 nm, 527 nm, 594 nm, 606 nm, 645 nm, 684 nm, 706 nm and 628 nm originate from P0 3→H4 3, P0 3→H5 3, D2 1→H4 3, P0 3→H6 3, P0 3→F2 3, D2 1→H5 3, P0 3→F3 3 и P0 3→F4 3 transitions of the Pr^3+^ ion, respectively. The presence of highly intensive ligand fluorescence in the tris complex spectrum indicates an incomplete transfer of energy to the excited states of the ion. Notably, there is a decrease in the relative intensity of the luminescence of the ligand for the tetrakis complex in comparison with the tris complex.

The spectra of both complexes of the Pr^3+^ ion are similarly split by the Stark effect, suggesting the same polyhedron symmetry for both compounds. This conclusion was supported by the X-ray single crystal structure analysis (D_4d_ group). Unfortunately, we could not obtain the spectrum in the NIR region for the tris complex due to intensive vibration quenching on the OH groups.

**Figure 8:**
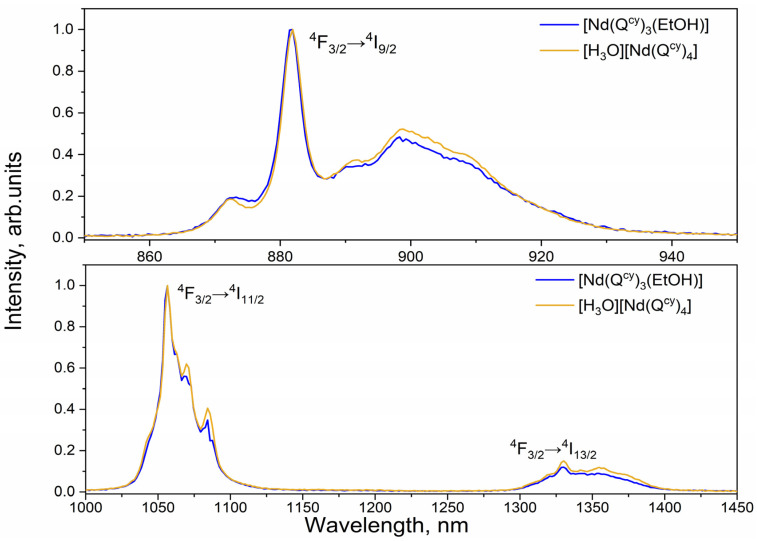
Photoluminescence spectra for the Nd^3+^ ion compounds in solid state under CW excitation at 340 nm.

The photoluminescence spectra of [Nd(Q^cy^)_3_(H_2_O)(EtOH)]∙(EtOH) and [H_3_O][Nd(Q^cy^)_4_] complexes are shown in Figure 8. The typical spectral bands for Nd^3+^ ions are observed in the spectra: F3/24→I9/2 4 880 nm, F3/24→I11/2 4 1056 nm и F3/24→I13/2 4 1330 nm. The emission bands related to F3/24→I9/2 4 880 nm, and F3/24→I11/2 4 electronic transitions are split by several Stark components centered at the same wavelengths for both the compounds. Therefore, there is no coordination polyhedron symmetry difference for these complexes.

### 2.4. Photoluminescence Excitation

**Figure 9:**
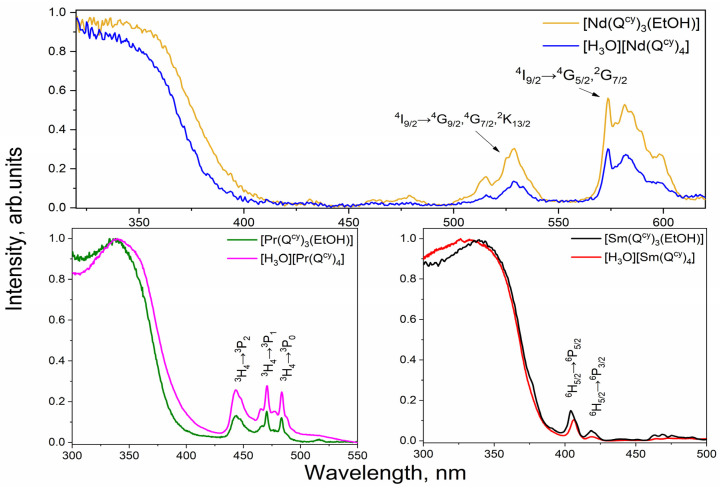
PL excitation spectra for compounds at solid state with registration wavelengths at PL intensity maxima.

The photoluminescence excitation spectra were obtained for all the investigated complexes with the registration wavelength of 1064 nm for Nd^3+^ ion complexes, 650 nm for Sm^3+^ ion complexes and 605 nm for Pr^3+^ ion complexes. The excitation spectra for tris and tetrakis complexes qualitatively resemble each other pairwise (See Figure 9). The most intensive excitation for all the complexes can be achieved via the ligand excited states with the maximum located at 340 nm. However, we also observed narrow excitation bands corresponding to resonant excitation of the ions through f-f* electronic transitions. Notably, the [Nd(Q^cy^)_3_(H_2_O)(EtOH)]∙(EtOH) complex has more intensive bands related to excitation through the ion than [H_3_O][Nd(Q^cy^)_4_], while for Pr^3+^ ion complexes, tetrakis one has more intensive bands related to excitation through the ion.

### 2.5. Judd–Ofelt Analysis

All the complexes were successfully investigated in terms of the Judd–Ofelt theory [47,48] and their Ω_t_ (t = 2, 4, 6) intensity parameters as well as the t_rad_ radiative lifetimes of the emission states of the ions are presented in Table 1, Table 2 and Table 3 and Tables S3–S5. The general procedure for Nd^3+^ and Sm^3+^ complexes was the same as in [2,20,49]. The calculations included in the transition bands are labeled in the absorption spectra of the complexes according to [44,50], see Figures S1–S3. The refractive index was set as 1.47 [51]. Notably, we carried out an analysis for praseodymium complexes via the standard JO theory with physically valid intensity parameters. The set of transitions used for the calculations was mostly chosen to minimize the root-mean-square deviation, see Tables S3–S5.

Radiative lifetimes have an increasing trend for the samarium and praseodymium tetraxis complexes. The strongest effect observed for the samarium complexes since t_rad_ for [H_3_O][Sm(Q^cy^)_4_] is 6 times higher than that for [Sm(Q^cy^)_3_(EtOH)]. Increasing of the radiative lifetime generally and mostly resulted from the stronger mixing of the 4f-states with the opposite parity states from configurations with higher energies [47]. Additionally, it is commonly considered that the parameter Ω_2_ is strongly enhanced by covalent bonding and depends on the degree of symmetry of the ligand environment. Relative to the analysis of the Stark splitting and X-ray analysis for these complexes (see one for the tris complex here [20]), we hardly observed any significant changes in symmetry or coordination number, but the maxima of the NIR-luminescent bands were slightly shifted for [Sm(Q^cy^)_3_(H_2_O)(EtOH)]∙(EtOH) indicating the so-called “nephelauxetic effect” [52]. This peculiarity is possibly related ton the five-times-greater value of Ω_2_ for the tris complex. Additionally, to the luminescent spectra of the complexes, evaluated branching ratio values for ^4^G_5/2_-^6^H_9/2_ transition are higher than 50% for both complexes, allowing us to consider them potentially suitable for lasing.

As for the praseodymium complexes, we conclude that the radiative lifetime t_rad_ increased by about twice for tetraxis for all discussed emission layers, viz. ^3^P_0_, ^3^P_1_ and ^1^D_2_. Since transitions from different emission layers are overlapped and strong ligand-centered luminescence occurs, we can hardly distinguish the experimental branching ratios; however, the calculated ones show a change, with branching ratios increasing for transitions of higher energies for the [H_3_O][Pr(Q^cy^)_4_] complex.

The analysis of the neodymium complexes reveals a 22% decrease in lifetime from 255 to 198 µs for tetraxis complexes, and no changes in Ω_2_ parameters. Thus, the symmetry state for both complexes is the same or very close. We suppose that this behavior results from changes in the delocalization of the outer d-orbitals of ions caused by the ligand field (this is the nephelauxetic effect).

### 2.6. Luminescent Decays and Quantum Yields

**Figure 10:**
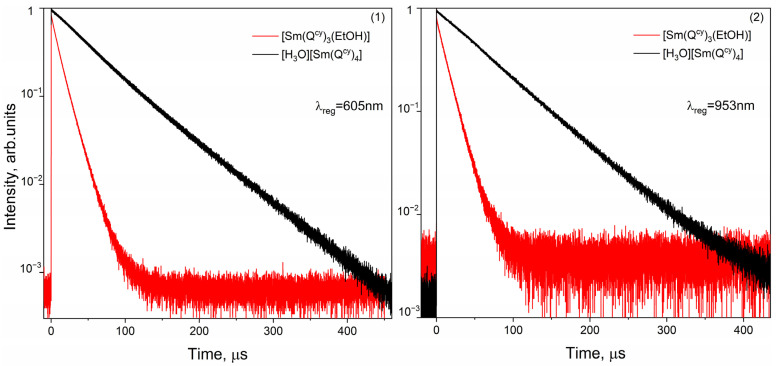
PL decays for the Sm^3+^ ion compounds in solid state under pulsed excitation of 355 nm monitored at the 650 nm (**1**) and 953 nm (**2**).

For a better understanding of electronic excitation and relaxation processes in the investigated compounds, luminescence decays were recorded. As seen in Figure 10, the tetrakis Sm^3+^ complex had a longer lifetime compared to the tris Sm^3+^ complex. In particular, characteristic lifetime measured at the registration wavelength of 650 nm for [H_3_O][Sm(Q^cy^)_4_] was 55 μs, which is four times greater than the lifetime of 13 μs recorded for [Sm(Q^cy^)_3_(H_2_O)(EtOH)]∙(EtOH). Similarly, an increase in the luminescence lifetime was observed in the NIR region with the registration at 953 nm (G5/2 4→F5/2 6) from 14 to 66 μs for tris and tetrakis compounds. Both complexes show monoexponential behavior at visible and NIR regions, which evidences that there is only one emission center.

**Figure 11:**
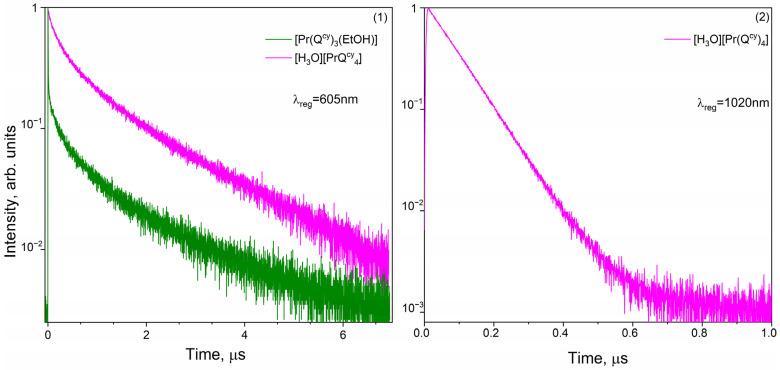
PL decays for the Pr^3+^ ion compounds in solid state under pulsed excitation of 355 nm monitored at 605 nm (**1**) and 1020 nm (**2**).

Luminescent decays for [Pr(Q^cy^)_3_(H_2_O)(EtOH)]∙(EtOH) and [H_3_O][Pr(Q^cy^)_4_] complexes are shown in Figure 11. Herein, the decays have a more complicated behavior than Sm^3+^ complexes and can be fitted by a multiexponential law:

(1) $$I_{th}\left(t\right)=\sum_{i=1}^{n}A_{i}e^{-\frac{t}{\tau_{i}}},$$

where *τ_i_* and *A_i_* are lifetimes and corresponding amplitudes, respectively. The measured luminescence decay is determined by

(2) $$I_{exp}\left(t\right)=\int_{0}^{\infty }I_{irf}\left(t^{\prime }\right)I_{th}\left(t-t^{\prime }\right)dt^{\prime },$$

where $I_{irf}\left(t^{\prime }\right)$ is the instrument response function (IRF), which can be described as a double-Gaussian function with the characteristic time *τ_irf_* = 0.5 ns for the measurements in the NIR region of the spectrum and an exponential function with the characteristic time *τ_irf_* =1 ns (see Figure S1 in Supplementary Material).

The Pr^3+^ ion complex decays obey a three-exponential law with characteristic lifetimes of several nanoseconds ($\tau_{1})$, hundreds of nanoseconds ($\tau_{2})$, and microseconds ($\tau_{3})$ (See Table 4). The longest lifetime $\tau_{3}$ was 1278 ns for [Pr(Q^cy^)_3_(H_2_O)(EtOH)]∙(EtOH) and 1693 ns for [H_3_O][Pr(Q^cy^)_4_], and is related to ligand phosphorescence, which was observed in PL spectra (See Figure 11). We could not measure the NIR tetrakis complex decay due to the extremely low luminescence intensity.

Specifically, the lifetimes obtained for [Pr(Q^cy^)_3_(H_2_O)(EtOH)]∙(EtOH) with registration at the visible spectral region are several times higher than the lifetimes at the NIR spectral region. This phenomenon can be explained by the fact that the radiative transition D2 1→F4 3 (1020 nm) is more susceptible to non-radiative relaxation on the O-H vibrations of water molecules than the transition P0→ 3H6 3 (606 nm) due to a small energy difference. Shorter lifetimes were observed for tris compounds ($606\;nm:\;\tau_{1}=5\;ns,\;\tau_{2}=382\;ns;1020\;nm:\tau_{1}=5\;ns,\;\tau_{2}=58\;ns$), compared to tetrakis compounds ($606\;nm:\;\tau_{1}=86\;ns,\;\tau_{2}=183\;ns;1020\;nm:\tau_{1}=85\;ns$). Notably, the [H_3_O][Pr(Q^cy^)_4_] complex had a monoexponential decay at the NIR spectral region, and the long-term decay component vanished, in contrast to [Pr(Q^cy^)_3_(H_2_O)(EtOH)]∙(EtOH) complex, which remained biexponential.

**Figure 12:**
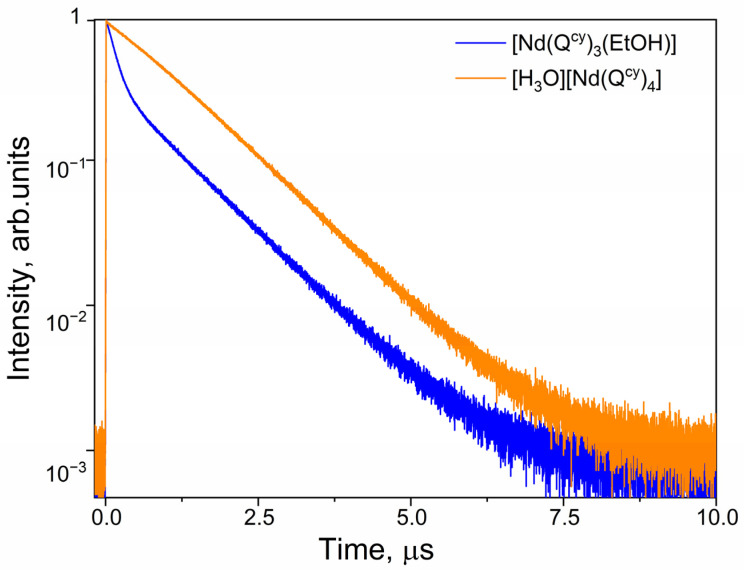
PL decays for the Nd^3+^ ion compounds in solid state under pulsed excitation of 355 nm monitored at the 1064 nm.

Photoluminescence decays for [Nd(Q^cy^)_3_(H_2_O)(EtOH)]∙(EtOH) and [H_3_O][Nd(Q^cy^)_4_] complexes, with the registration wavelength of 1056 nm, are shown in Figure 12. The tris complex has biexponentially fitted decay with characteristic lifetimes $\tau_{1}=146$ ns and $\tau_{2}=1046$ ns. The tetrakis complex has monoexponential behaviour, and its characteristic lifetime is τ=1183 ns.

Photoluminescence quantum yield (Φ) values were measured for all the compounds under optical excitation at 365 nm. As seen from Table 4, Nd^3+^ ion-based complexes have similar values of Φ = 1.3%, and Pr^3+^ ion based complexes have Φ = 0.4%. However, the [Sm(Q^cy^)_3_(H_2_O)(EtOH)]∙(EtOH) complex has Φ = 0.5% in the NIR spectral area and Φ = 1.3% in the visible spectral area, while [H_3_O][Sm(Q^cy^)_4_] complex has Φ = 0.4% and Φ = 2.0% in the NIR and visible regions, respectively. Therefore, adding the fourth ligand molecule to a compound leads to a significant increase in Φ for visible emissions, while there is no effect in the NIR region.

The ^4^G_5/2_ → ^6^H_9/2_ transition of the Sm^3+^ ion, which is the most intensive emission band, is an electric dipole transition, while the ^4^G_5/2_ → ^6^H_5/2_ transition is a magnetic dipole transition. On that basis, ligand environment polarizability of Sm^3+^ ion tris and tetrakis complexes was estimated as the difference between the integrated magnetic dipole and electro dipole transitions. As we observed a bigger value for the tetrakis complex (10.3 for tetrakis against 7.8 for tris), we conclude that it is more polarized, which can explain the increase in the quantum yield in the visible region for the tetrakis complex in comparison with the tris one.

## 3. Materials and Methods

### 3.1. Experimental Setups

Single-crystal X-ray diffraction analysis of [Ln(Q^cy^)_3_(H_2_O)(EtOH)]∙(EtOH) (Ln = Sm, Pr, Nd) was carried out on a Bruker D8 Venture diffractometer (MoK_α_ radiation, ω and φ-scan mode), and SCXRD analysis of (H_3_O)^+^[Ln(Q^cy^)_4_]^−^ (Ln = Sm, Pr, Nd) was carried out on a Bruker D8 Quest diffractometer (MoK_α_ radiation, ω and φ-scan mode). The structures were solved with direct methods and refined using the least-squares method in the full-matrix anisotropic approximation on F^2^. All hydrogen atoms were located in calculated positions and refined within a riding model. All calculations were performed using the SHELXTL [53,54] and Olex2 [55] software packages. Atomic coordinates, bond lengths, angles, and thermal parameters have been deposited at the Cambridge Crystallographic Data Centre with deposition numbers—CCDC 2280580-2280585, which are available free of charge at www.ccdc.cam.ac.uk (accessed on 13 July 2023).

Elemental analysis was performed on an Elementar VarioEL Cube CHNO(S) analyzer (Ele-mentar Analysensysteme GmbH, Langenselbold, Germany). The lanthanide content was determined by complexometric titration with a standard Trilon B (disodium salt of ethylenediaminetetraacetic acid) solution in the presence of Xylenol Orange as an indicator. The sample was decomposed by heating with 70% HNO_3_ before titration [56].

Visible and NIR absorption spectra for all the complexes and free ligands were recorded on a JASCO V-770 (Jasco, Tokyo, Japan) spectrophotometer operating within 200–3200 nm. Concentrations of the solutions were approximately 10^−5^ M. For solutions, the measurements were performed using quartz cells with a 1 cm pathlength.

Visible photoluminescence spectra and excitation spectra for the complexes and the free ligand were obtained at room temperature using a Horiba Jobin-Yvon Fluorolog QM-75-22-C spectrofluorimeter with a 75 W xenon arc lamp (PowerArc, HORIBA, Kyoto, Japan). A Hamamatsu R13456 (Hamamatsu Photonics, Hamamatsu, Japan) cooled photomultiplier tube sensitive in the UV–Vis–NIR region (200–950 nm) was used as the detector. For the NIR spectral region measurements, the same setup was used, except for the detector, which was replaced by a Hamamatsu H10330 cooled photomultiplier tube sensitive in NIR region (950–1700 nm). Luminescence decays in the visible region and NIR region of the spectrum for the complexes were obtained in solid state using the same setup equipped with a Xe flash lamp as the excitation source.

Luminescence quantum yields in the visible region were obtained using an absolute method with by a home-made setup based on a MgO-covered integrating sphere with a diameter of 180 mm and FD-10G calibrated germanium photodiode detector; a CW emitting LED (365 nm) was used as an excitation source. Each sample was measured a few times under slightly different experimental conditions, and the results were averaged. The estimated error for the quantum yields was ±10%.

For all optical measurements, the corresponding instrument response functions were taken into account. The experiments were performed in air at atmospheric pressure. Degradation of the optical properties was not observed during the experiments.

### 3.2. Synthesis

Commercially available reagents and solvents were used for synthesis without further purification unless otherwise stated. The HQ^cy^ ligand was synthesized according to the previously described method [57]. Lanthanide chloride crystalline hydrates were obtained by dissolving the corresponding oxides (99.999%, LANHIT, Moscow, Russia) in extra pure hydrochloric acid.

The tetrakis complexes H_3_O[Ln(Q^cy^)_4_] were prepared according to the previously reported procedure [3,20]. For IR spectra see Figure S4. The powder XRD method was used to confirm that the powder phase composition corresponds to a single crystal (see Figure S5).

H_3_O[Pr(Q^cy^)_4_], 1. Green plates, 75%. Anal. Calc. for C_68_H_79_N_8_O_9_Pr: C, 63.15; H, 6.16; N, 8.65; Pr, 10.89%. Found: C, 62.1; H, 6.4; N, 8.6; Pr, 10.5. IR (cm^−1^): 3510 VW (ν O–HH_3_O+), 2927 M, 2851 W (ν C-HCyclohexyl), 1672 W (δ O–HH_3_O+), 1614 VS (ν C=ODiketone), 1594 S, 1581 M, 1497 VS (ν C–C), 1484 S, 1461 M, 1437 S, 1410 M, 1399 M, 1369 M, 1324 W, 1149 VW, 1124 VW, 1079 M, 1029 W, 1009 W, 999 W, 978 M, 921 W, 906 W, 809 W.

H_3_O[Nd(Q^cy^)_4_], 1. Pale violet plates, 74%. Anal. Calc. for C_68_H_79_N_8_NdO_9_: C, 62.99; H, 6.14; N, 8.64; Nd, 11.12%. Found: C, 63.1; H, 6.7; N, 8.5; Nd, 10.5. IR (cm^−1^): 3604 VW (ν O–HH_3_O+), 2926 S, 2851 S (ν C-HCyclohexyl), 1672 S (δ O–HH_3_O+), 1615 VS (ν C=ODiketone), 1594 S, 1581 S, 1498 VS (ν C–C), 1484 VS, 1461 S, 1437 VS, 1410 S, 1399 S, 1368 VS, 1324 S, 1234 M, 1149 M, 1079 S, 1029 M, 1009 M, 999 M, 978 S, 920 W, 904 M, 809 S, 691 S.

H_3_O[Sm(Q^cy^)_4_], 1. Pale yellow plates, 78%. Anal. Calc. for C_68_H_79_N_8_O_9_Sm: C, 62.69; H, 6.11; N, 8.60; Sm, 11.54%. Found: C, 62.0; H, 6.7; N, 8.5; Sm, 11.9. IR (cm^−1^): 3508 VW (ν O–HH_3_O+), 2927 M, 2852 M (ν C-HCyclohexyl), 1674 M (δ O–HH_3_O+), 1616 VS (ν C=ODiketone), 1594 S, 1582 S, 1498 VS (ν C–C), 1486 S, 1461 S, 1411 M, 1399 M, 1370 S, 1324 W, 1234 W, 1150 W, 1125 W, 1080 S, 1029 M, 1009 W, 999 W, 978 M, 921 W, 905 W, 904 W, 810 M.Tris complexes [Ln(Q^cy^)_3_(H_2_O)(EtOH)]∙(EtOH). were synthesized using a modified method [3]. For IR spectra see Figure S4. The powder XRD method was used to confirm that the powder phase composition corresponds to a single crystal (see Figure S6).

[Pr(Q^cy^)_3_(H_2_O)(EtOH)]∙(EtOH) Green needles, 66%. Anal. Calc. for C_55_H_71_N_6_O_9_Pr: C, 59.99; H, 6.50; N, 7.61; Pr, 12.80%. Found: C, 60.1; H, 6.5; N, 7.6; Pr, 12.8. IR (cm^−1^): 3590 VW (ν O–H H_2_O), 2929 M, 2852 W (ν C-HCyclohexyl), 1646 M (δ O–H H_2_O), 1614 VS (ν C=ODiketone), 1593 S, 1582 S, 1531 M, 1499 S (ν C–C), 1460 S, 1453 M, 1437 S, 1398 M, 1371 M, 1325 W, 1234 W, 1200 VW, 1176 VW, 1156 VW, 1142 VW, 1129 VW, 1079 M, 1027 W, 1010 W, 1000 W, 979 M, 905 VW, 756 M.

[Nd(Q^cy^)_3_(H_2_O)(EtOH)]∙(EtOH) Pale violet needles, 66%. Anal. Calc. for C_55_H_71_N_6_O_9_Nd: C, 59.81; H, 6.48; N, 7.61; Nd, 13.06%. Found: C, 60.2; H, 6.5; N, 7.6; Nd, 13.1. IR (cm^−1^): 3632 VW (ν O–H H_2_O), 2930 M, 2852 W (ν C-HCyclohexyl), 1645 M (δ O–H H_2_O), 1613 VS (ν C=ODiketone), 1594 S, 1582 S, 1532 M, 1499 S (ν C–C), 1461 S, 1453 M, 1438 S, 1399 M, 1374 M, 1325 W, 1235 W, 1201 VW, 1176 VW, 1157 VW, 1142 VW, 1129 VW, 1079 M, 1028 W, 1010 W, 1000 W, 979 M, 756 M.

[Sm(Q^cy^)_3_(H_2_O)(EtOH)]∙(EtOH) Pale yellow needles, 62%. Anal. Calc. for C_55_H_71_N_6_O_9_Sm: C, 59.48; H, 6.44; N, 7.57; Sm, 13.54%. Found: C, 59.7; H, 6.5; N, 7.6; Sm, 13.7. IR (cm^−1^): 3627 VW (ν O–HH_3_O+), 2930 M, 2852 W (ν C-HCyclohexyl), 1672 VW (δ O–HH_3_O+), 1614 VS (ν C=ODiketone), 1594 S, 1582 M, 1532 M, 1501 VS (ν C–C), 1487 S, 1461 M, 1451 S, 1437 S, 1398 M, 1373 M, 1324 W, 1234 W, 1176 W, 1157 W, 1129 W, 1079 M, 1027 W, 1010 W, 1000 W, 979 M, 921 W, 755 W.

## 4. Conclusions

Tris and tetrakis coordination compounds of Sm^3+^, Nd^3+^ and Pr^3+^ ions were investigated pairwise in the present work. We found that adding an additional ligand molecule significantly increases the molar extinction by up to two times. In addition, it slightly changes the shapes of the Pr^3+^ and Sm^3+^ complexes’ PL spectra in the visible region and strongly affects the shape of the Pr^3+^ ion tetrakis complex PL spectrum in the NIR region. However, the polyhedron symmetry does not alter as a result of adding a fourth ligand molecule, except for Sm^3+^ complexes, where we observe lower symmetry for the tetrakis complex than for the tris complex. We also report a significant difference in luminescence lifetime for tris and tetrakis complexes of Sm^3+^ and Nd^3^ ions. Notably, Sm^3+^ tris and tetrakis complexes have similar luminescence decay behavior in the visible and NIR regions pairwise. Conversely, the short-time component disappears for the tetrakis Pr^3+^ complex in the NIR region.

While Nd^3+^ and Pr^3+^ ions complexes have the same quantum yield values of 1.3% and 0.4% (in visible region), respectively, we observed a huge increase in quantum yield for the Sm^3+^ tetrakis complex in the visible region in comparison with the tris complex (from 1.3% to 2.0%), although their quantum yield values in NIR region are practically equal (0.4–0.5%). We consider that the luminescence quantum yield increase in the Sm^3+^ ion complexes is related not only to the substitution of water molecules by a ligand molecule, but also to the polyhedron symmetry change.

The findings of this study may have significant implications in optoelectronics, telecommunication, and bioimaging, where the utilization of luminescence in the NIR region of the spectrum can lead to advancements in sensing, imaging, and other cutting-edge technologies.

## Figures and Tables

**Table 1:** Calculated electric-dipole transition probabilities A_rad_, branching ratios b_calc_, and radiative lifetime t_rad_ for praseodymium complexes.

		[Pr(Q^cy^)_3_(H_2_O)(EtOH)]∙(EtOH)		[H_3_O][Pr(Q^cy^)_4_]	
^3^P_0_ ⟶ ^2S+1^L_J_	Wavelength, nm	A_rad_, s^−1^	b_calc_, %	A_rad_, s^−1^	b_calc_, %
^1^D_2_	3051	26.4	0.03	8.5	0.02
^3^F_4_	697	1165.5	1.6	1996.7	5.4
^3^F_2_	635	56,964.5	81.1	18,349.5	49.7
^3^H_6_	608	6415.1	9.1	6886.3	18.6
^3^H_4_	482	5656.8	8.1	9690.4	26.2
		t_rad_ = 14.2 µs;		t_rad_ = 27.1 µs;	
^3^P_1_ ⟶ ^2S+1^L_J_	Wavelength, nm	A_rad_, s^−1^	b_calc_, %	A_rad_, s^−1^	b_calc_, %
^1^D_2_	2223	359.7	0.2	115.9	0.1
^3^F_4_	688	2973.2	1.5	5093.23	4.7
^3^F_3_	669	97,011.6	49.2	34,600.8	31.8
^3^F_2_	611	58,151.7	29.5	18,731.9	17.3
^3^H_6_	586	12,305.9	6.2	13,209.9	12.2
^3^H_5_	520	20,134.5	10.2	26,390.4	24.3
^3^H_4_	468	6083.7	3.08	10,421.8	9.6
		t_rad_ = 5.1 µs;		t_rad_ = 9.2 µs;	
^1^D_2_ ⟶ ^2S+1^L_J_	Wavelength, nm	A_rad_, s^−1^	b_calc_, %	A_rad_, s^−1^	b_calc_, %
^3^F_4_	995	30,580.5	74.6	10,155.6	51.9
^3^F_3_	956	1880.1	4.6	708.9	3.6
^3^F_2_	843	1685.3	4.1	1279.6	6.5
^3^H_6_	796	757.8	1.8	1131.9	5.8
^3^H_5_	678	47.9	0.1	65.7	0.3
^3^H_4_	592	6048.3	14.8	6206.9	31.8
		t_rad_ = 24.3 µs;		t_rad_ = 51.2 µs;	

**Table 2:** Calculated electric-dipole transition probabilities A_rad_, branching ratios b_calc_, and radiative lifetime t_rad_ for neodymium complexes.

		[Nd(Q^cy^)_3_(H_2_O)(EtOH)]∙(EtOH)		[H_3_O][Nd(Q^cy^)_4_]	
^4^F_3/2_ ⟶ ^2S+1^L_J_	Wavelength, nm	A_rad_, s^−1^	b_calc_, %	A_rad_, s^−1^	b_calc_, %
^4^I_15/2_	880	1890.2	48.2	2951.6	52.6
^4^I_13/2_	1060	1717.7	43.8	2056.5	40.8
^4^I_11/2_	1330	295.9	7.5	314.9	6.2
^4^I_9/2_	1830	15.2	0.4	16.2	0.3
		t_rad_ = 255.2 µs;		t_rad_ = 198.5 µs;	

**Table 3:** Calculated electric-dipole transition probabilities A_rad_, branching ratios b_calc_, and radiative lifetime t_rad_ for samarium complexes.

		[Sm(Q^cy^)_3_(H_2_O)(EtOH)]∙(EtOH)		[H_3_O][Sm(Q^cy^)_4_] *	
^4^G_5/2_ ⟶ ^2S+1^L_J_	Wavelength, nm	A_rad_, s^−1^	b_calc_, %	A_rad_, s^−1^	b_calc_, %
^6^H_5/2_	565	42.4	2.5	8.6	2.7
^6^H_7/2_	610	201.4	12.1	46.2	14.9
^6^H_9/2_	650	1006.7	60.4	178.7	57.6
^6^H_11/2_	715	57.1	3.4	14.3	4.6
^6^H_13/2_	800	5.8	0.3	0.8	0.3
^6^F_3/2_	936	36.3	2.2	6.1	1.9
^6^F_5/2_	949	202.8	12.1	34.9	11.2
^6^F_7/2_	1036	5.5	0.3	1.5	0.5
^6^F_9/2_	1180	108.2	6.4	18.7	6.0
		t_rad_ = 0.6 ms;		t_rad_ = 3.2 ms;	

* Data were revealed previously in [20].

**Table 4:** Comparison of characteristic lifetimes of coordination compounds on different registration wavelengths.

Complex	λ, nm	$\tau_{1}$, ns	$\tau_{2}$, ns	Φ, %
[Nd(Q^cy^)_3_(H_2_O)(EtOH)]∙(EtOH)	1056	146	1046	1.3
$\left[H_{3}O\right]\left[Nd{\left(Q^{cy}\right)}_{4}\right]$	1056	1183	–	1.3
[Pr(Q^cy^)_3_(H_2_O)(EtOH)]∙(EtOH)	605	5	184	0.4
	1020	5	58	–
$\left[H_{3}O\right]\left[Pr{\left(Q^{cy}\right)}_{4}\right]$	605	81	392	0.4
	1020	85	–	–
[Sm(Q^cy^)_3_(H_2_O)(EtOH)]∙(EtOH)	650	13 × 10^3^	–	1.3
	953	14 × 10^3^	–	0.5
$\left[H_{3}O\right]\left[Sm{\left(Q^{cy}\right)}_{4}\right]$	650	55 × 10^3^	–	2.0
	953	66 × 10^3^	–	0.4

## Data Availability

Data available upon request.

## Supplementary Materials

The following supporting information can be downloaded at https://www.mdpi.com/article/10.3390/molecules28155892/s1, Figure S1: Absorption spectra for praseodymium complexes in DMSO solvent with concentration of 3 × 10^−3^ M.; Figure S2: Absorption spectra for neodymium complexes in DMSO solvent with concentration of 3 × 10^−3^ M.; Figure S3: Absorption spectra for samarium complexes in DMSO solvent with concentration of 3 × 10^−3^ M.; Figure S4: IR transmittance spectra of complexes (KBr pellets, 298K).; Figure S5: PXRD patterns of tetrakis-complexes of Pr, Nd and Sm and simulated from single crystal data of Sm complex. Figure S6: PXRD patterns of tris-complexes of Pr, Nd and Sm and simulated from single crystal data of Pr complex. Table S1: Crystal data and refinement parameters for [Sc(Q^cy^)_3_(DMSO)], [La(Q^cy^)_3_(H_2_O)(EtOH)]∙(EtOH), [Gd(Q^cy^)_3_(H_2_O)] and [Lu(Q^cy^)_3_(DMSO)]; Table S2: C–O and C–C bond lengths of the diketone fragment in (H_3_O)^+^[Sm(Q^cy^)_4_]^−^; Table S3: Experimental f_exp_ and calculated f_calc_ oscillator strengths, Judd–Ofelt parameters Ω_t_ (t = 2, 4, 6), and root-mean-squared deviation RMS for praseodymium complexes; Table S4: Experimental f_exp_ and calculated f_calc_ oscillator strengths, Judd–Ofelt parameters Ω_t_ (t = 2, 4, 6), and root-mean-squared deviation RMS for neodymium complexes; Table S5: Experimental f_exp_ and calculated f_calc_ oscillator strengths, Judd–Ofelt parameters Ω_t_ (t = 2, 4, 6), and root-mean-squared deviation RMS for samarium complexes.
