# Supplementary material for: Influence of Ligand Environment Stoichiometry on NIR-Luminescence Efficiency of Sm3+, Pr3+ and Nd3+ Ions Coordination Compounds
Source: Molecules. 2023 Aug 5;28(15):5892. doi: 10.3390/molecules28155892 (PMC10421502; doi:10.3390/molecules28155892)
Supplement: Supplementary file 1 [file molecules-28-05892-s001.zip › molecules-2533269-supplementary.pdf]

Influence of ligand environment stoichiometry on NIR-luminescence efficiency of Sm^{3+}, Pr^{3+} and Nd^{3+} ions coordination compounds

Supporting information

Table S1. Crystal data and refinement parameters for $[Sc(Q^{cy})_3(DMSO)]$, $[La(Q^{cy})_3(H_2O)(EtOH)]\cdot(EtOH)$, $[Gd(Q^{cy})_3(H_2O)]$ and $[Lu(Q^{cy})_3(DMSO)]$.

Parameter	Value					
	$[Pr(Q^{CH})_3(H_2O)(EtOH)_{0.5}(MeOH)_{0.5}]\cdot(EtOH)$	$[Nd(Q^{CH})_3(H_2O)(EtOH)_{0.65}(MeOH)_{0.35}]\cdot(EtOH)$	$[Sm(Q^{CH})_3(H_2O)(EtOH)]\cdot(EtOH)$	$(H_3O)^+[Pr(Q^{CH})_4]^-$	$(H_3O)^+[Nd(Q^{CH})_4]^-$	$(H_3O)^+[Sm(Q^{CH})_4]^-$
Molecular Formula	$C_{54.50}H_{69.50}N_6O_9Pr$	$C_{54.65}H7_{0.30}N_6NdO_9$	$C_{55}H_{71}N_6O_9Sm$	$C_{68}H_{79}N_8O_9Pr$	$C_{68}H_{79}N_8NdO_9$	$C_{68}H_{79}N_8O_9Sm$
M	1093.57	1099.50	1110.52	1293.30	1296.63	1302.74
Temperature, K	296(2)	293(2)	296(2)	100(2)	100(2)	100(2)
System	Monoclinic	Monoclinic	Monoclinic	Monoclinic	Monoclinic	Monoclinic
Space group	*C*2/*c*	*C*2/*c*	*C*2/*c*	*C*2/*c*	*C*2/*c*	*C*2/*c*
a, Å	18.593(9)	18.629(7)	18.5558(6)	14.9053(6)	14.8947(5)	14.8405(7)
b, Å	21.678(11)	21.651(17)	21.4507(7)	25.0384(10)	25.0567(7)	25.1013(14)
c, Å	27.160(11)	27.197(12)	27.0297(9)	16.5415(7)	16.5618(5)	16.5864(7)
α, deg.	90	90	90	90	90	90
β, deg.	102.807(14)	102.604(13)	102.3750(10)	93.3300(16)	93.4200(13)	93.660(2)
γ, deg.	90	90	90	90	90	90
V, $Å^3$	10675(9)	10705(10)	10508.8(6)	6162.9(4)	6170.1(3)	6166.1(5)
Z	8	8	8	4	4	4
ρ_{calc}, g/cm^3	1.361	1.364	1.404	1.394	1.396	1.403
$\mu(MoK_\alpha)$, mm^{-1}	0.973	1.030	1.179	0.855	0.906	1.017
F(000)	4556	4578	4616	2696	2700	2708
θmin–θmax, deg.	2.30 - 29.56	2.29 – 28.70	2.29 – 28.00	1.59 – 30.00	2.59 – 28.00	1.60 – 27.00
Number of measured reflections	61072	58000	51754	32457	27509	25838
Number of unique reflections (R_{int})	14956 (0.0351)	13832 (0.0427)	12685 (0.0319)	8996 (0.0577)	7458 (0.0481)	6716 (0.0792)
Number of reflections with $I > 2\sigma(I)$	13186	12306	11635	8037	6907	5786
Number of refined parameters	672	666	664	432	441	441
R-factors ($I > 2\sigma(I)$)	R_1 = 0.0449, ωR_2 = 0.0930	R_1 = 0.0494, ωR_2 = 0.0927	R_1 = 0.0450, ωR_2 = 0.0898	R_1 = 0.0608, ωR_2 = 0.1439	R_1 = 0.0537, ωR_2 = 0.1265	R_1 = 0.0581, ωR_2 = 0.1252
R-factors (all reflections)	R_1 = 0.0531, ωR_2 = 0.0959	R_1 = 0.0574, ωR_2 = 0.0950	R_1 = 0.0504, ωR_2 = 0.0915	R_1 = 0.0684, ωR_2 = 0.1486	R_1 = 0.0583, ωR_2 = 0.1295	R_1 = 0.0681, ωR_2 = 0.1313
GOOF	1.150	1.246	1.284	1.108	1.108	1.077
$\Delta\rho_{max}$ / $\Delta\rho_{min}$, e/$Å^3$	1.272 / -1.400	1.029/ -1.474	2.275 / -1.547	2.011 / -1.316	2.057 / -0.778	1.096/ -0.897

Table S2. C-O and C-C bond lengths of the diketone fragment in $(H_3O)^+[Sm(Q^{cy})_4]^-$.

Bond	Bond length, Å	Bond length Δ, Å
O1-C1	1.278	0.022
O2-C5	1.256	
O3-C18	1.256	0,023
O4-C22	1.233	
C1-C2	1.425	0.01
C2-C5	1.415	
C18-C19	1.414	0.029
C19-C22	1.443	

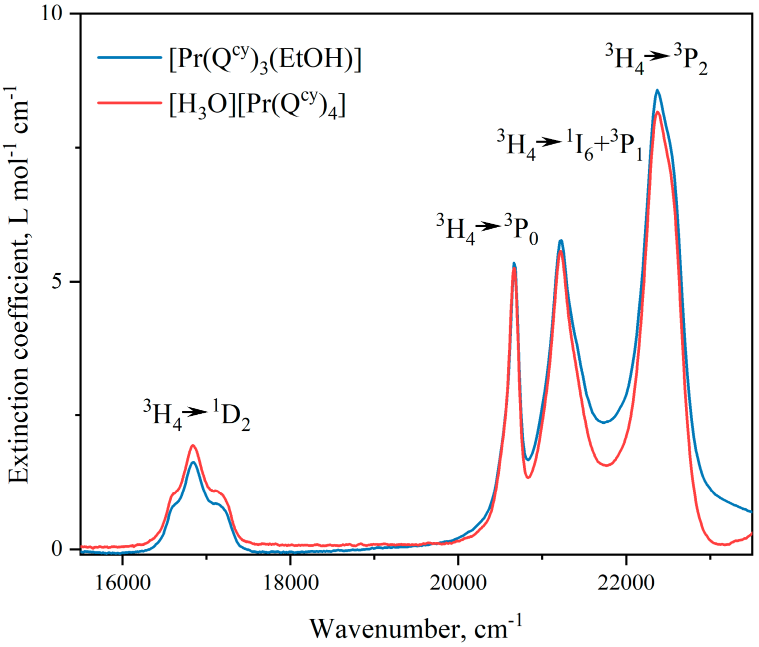

Figure S1. Absorption spectra for praseodymium complexes in DMSO solvent with concentration of $3 \cdot 10^{-3}$ M.

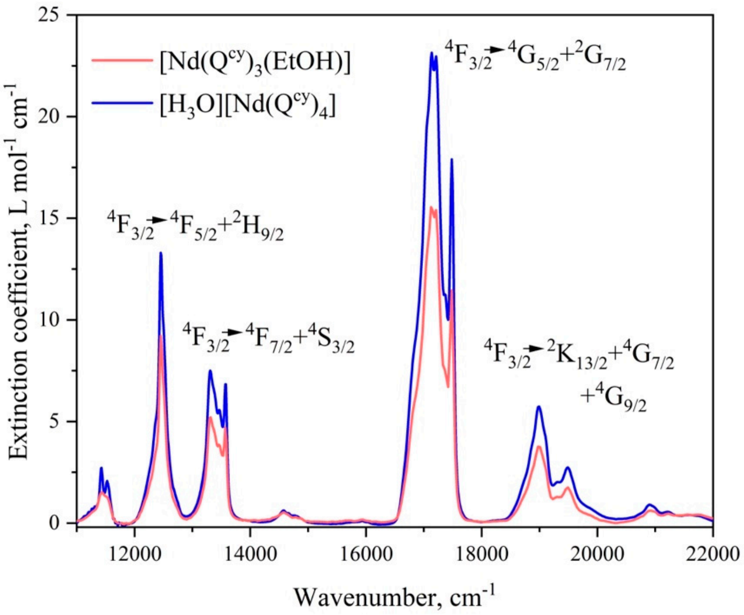

Figure S2. Absorption spectra for neodymium complexes in DMSO solvent with concentration of $3 \cdot 10^{-3}$ M.

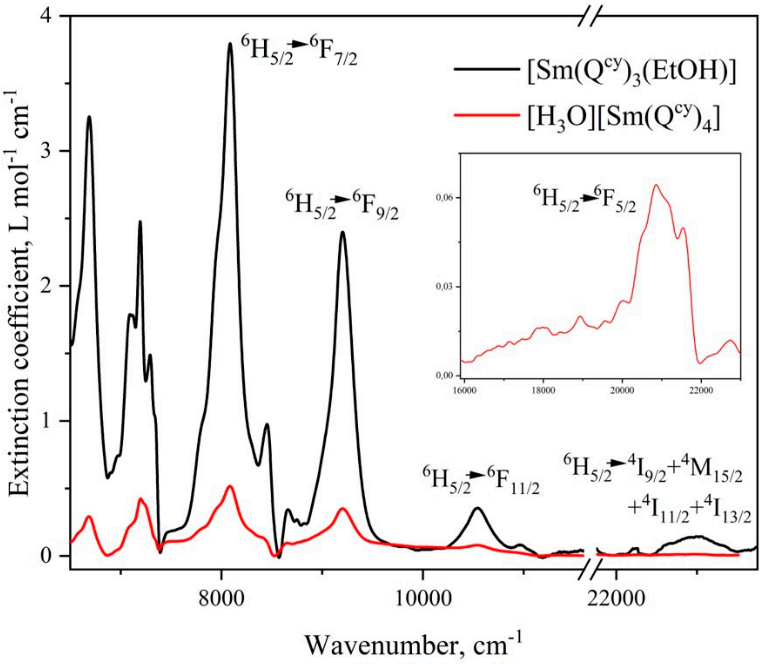

Figure S3. Absorption spectra for samarium complexes in DMSO solvent with concentration of $3\cdot10^{-3}$ M.

Table S3. Experimental f_{exp} and calculated f_{calc} oscillator strengths, Judd-Ofelt parameters Ω_t (t=2,4,6), and root-mean-squared deviation RMS for praseodymium complexes.

	$[Pr(Q^{cy})_3(H_2O)(EtOH)]\cdot(EtOH)$			$[H_3O][Pr(Q^{cy})_4]$		
Transition	Wavelength, nm	$f_{exp}\cdot10^6$	$f_{calc}\cdot10^6$	Wavelength, nm	$f_{exp}\cdot10^6$	$f_{calc}\cdot10^6$
$^3H_4 \rightarrow {}^3P_2$	446	18.0	17.6	447	19.8	19.4
$^3H_4 \rightarrow ({}^1I_6+{}^3P_1)$	469	10.8	10.5	471	12.5	12.1
$^3H_4 \rightarrow {}^3P_0$	483	3.2	3.6	484	5.8	6.2
$^3H_4 \rightarrow {}^1D_2$	593	4.8	5.6	594	4.8	5.7
RMS	$1.1\cdot10^{-6}$			$1.3\cdot10^{-6}$		
Ω_2, cm^2	$8.1\cdot10^{-19}$			$2.6\cdot10^{-19}$		
Ω_4, cm^2	$0.6\cdot10^{-19}$			$1.0\cdot10^{-19}$		
Ω_6, cm^2	$3.2\cdot10^{-19}$			$3.5\cdot10^{-19}$		

Table S4. Experimental f_{exp} and calculated f_{calc} oscillator strengths, Judd-Ofelt parameters Ω_t (t=2,4,6), and root-mean-squared deviation RMS for neodymium complexes.

	$[Nd(Q^{cy})_3(H_2O)(EtOH)]\cdot(EtOH)$			$[H_3O][Nd(Q^{cy})_4]$		
Transition	Wavelength, nm	$f_{exp}\cdot10^5$	$f_{calc}\cdot10^5$	Wavelength, nm	$f_{exp}\cdot10^5$	$f_{calc}\cdot10^5$
$^4I_{9/2} \rightarrow {}^4F_{5/2}+{}^2H_{9/2}$	804	0.8	0.9	802	1.2	1.3
$^4I_{9/2} \rightarrow {}^4F_{7/2}+{}^4S_{3/2}$	750	0.8	0.8	747	1.1	1.1
$^4I_{9/2} \rightarrow {}^4G_{5/2}+{}^2G_{7/2}$	583	1.0	0.9	581	1.5	1.3
$^4I_{9/2} \rightarrow {}^2K_{13/2}+{}^4G_{7/2}+{}^4G_{9/2}$	524	3.6	3.5	523	5.3	5.3
RMS	$9.5\cdot10^{-7}$			$2.3\cdot10^{-6}$		
Ω_2, cm^2	$9.0\cdot10^{-20}$			$8.9\cdot10^{-20}$		
Ω_4, cm^2	$8.0\cdot10^{-20}$			$1.4\cdot10^{-19}$		

Ω_6, cm^2	$5.5\cdot10^{-19}$	$6.9\cdot10^{-20}$

Table S5. Experimental f_{exp} and calculated f_{calc} oscillator strengths, Judd-Ofelt parameters Ω_t (t=2,4,6), and root-mean-squared deviation RMS for samarium complexes.

	$[Sm(Q^{cy})_3(H_2O)(EtOH)]\cdot(EtOH)$			$[H_3O][Sm(Q^{cy})_4]$*		
Transition	Wavelength, nm	$f_{exp}\cdot10^6$	$f_{calc}\cdot10^6$	Wavelength, nm	$f_{exp}\cdot10^8$	$f_{calc}\cdot10^8$
$^6H_{5/2} \rightarrow {}^6F_{7/2}$	1242	5.1	5.1	1237	99.4	99.4
$^6H_{5/2} \rightarrow {}^6F_{9/2}$	1084	3.1	3.1	1086	49.0	49.2
$^6H_{5/2} \rightarrow {}^6F_{11/2}$	949	0.5	0.5	948	8.5	7.4
$^6H_{5/2} \rightarrow ({}^4I_{9/2}+{}^4M_{15/2}+{}^4I_{11/2}+{}^4I_{13/2})$	475	1.9	1.9	-	-	-
$^6H_{5/2} \rightarrow {}^6F_{5/2}$	-	-	-	454	1.6	1.8
RMS	$3.2\cdot10^{-8}$			$1.3\cdot10^{-8}$		
Ω_2, cm^2	$4.7\cdot10^{-19}$			$8.1\cdot10^{-20}$		
Ω_4, cm^2	$6.8\cdot10^{-20}$			$1.8\cdot10^{-20}$		
Ω_6, cm^2	$3.7\cdot10^{-19}$			$0.5\cdot10^{-20}$		

*Data was revealed previously in [10.3390/polym15040867]

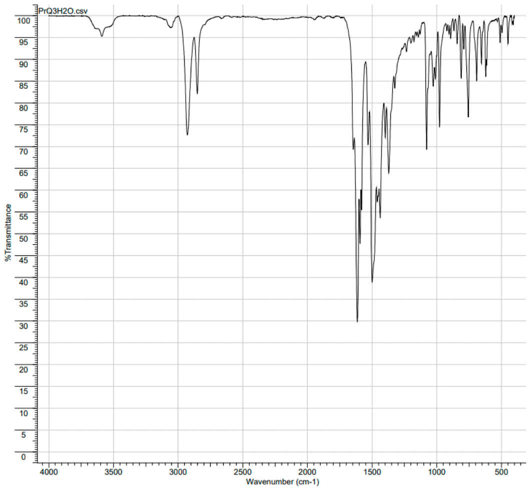

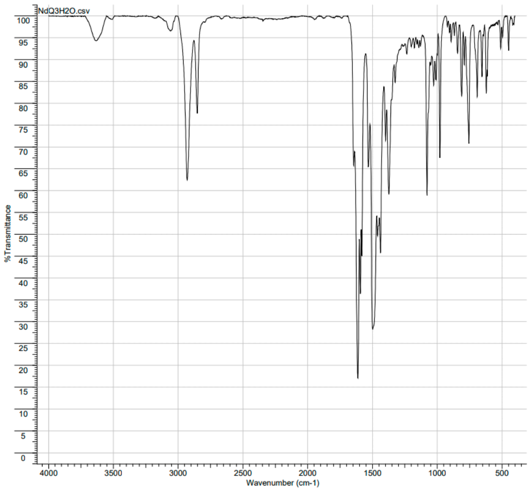

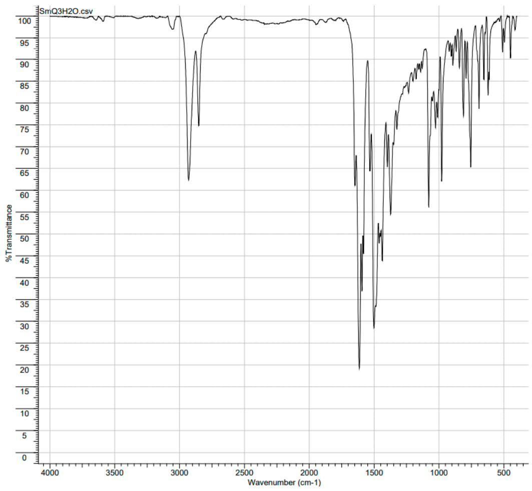

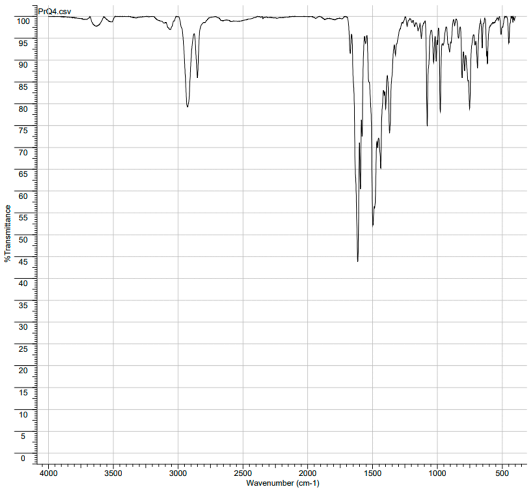

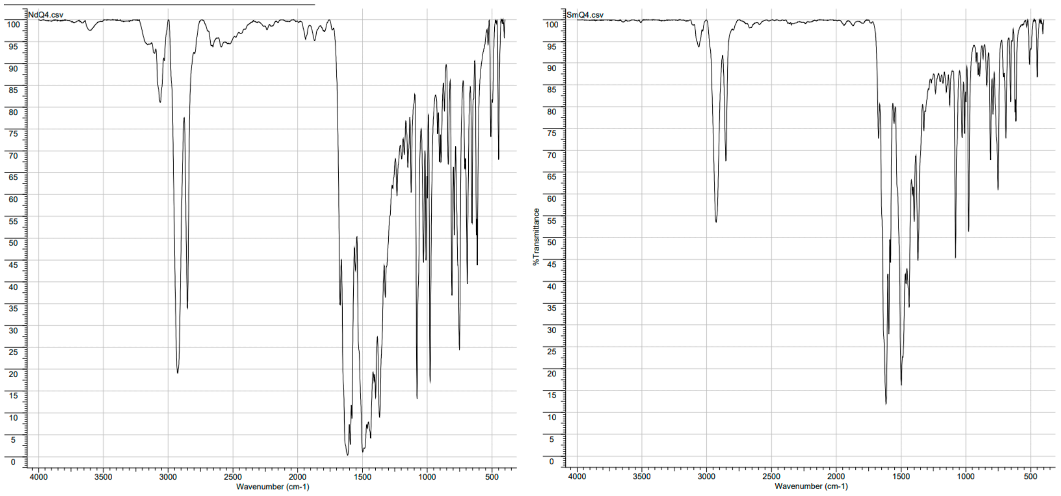

Figure S4. IR transmittance spectra of complexes (KBr pellets, 298K).

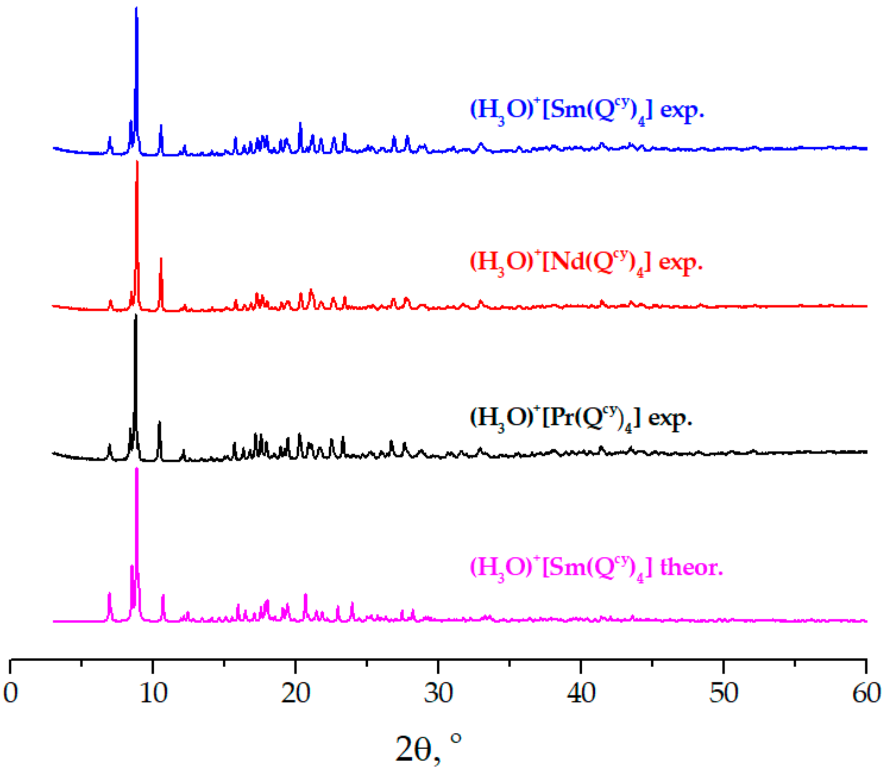

Figure S5. PXRD patterns of tetrakis-complexes of Pr, Nd and Sm and simulated from single crystal data of Sm complex.

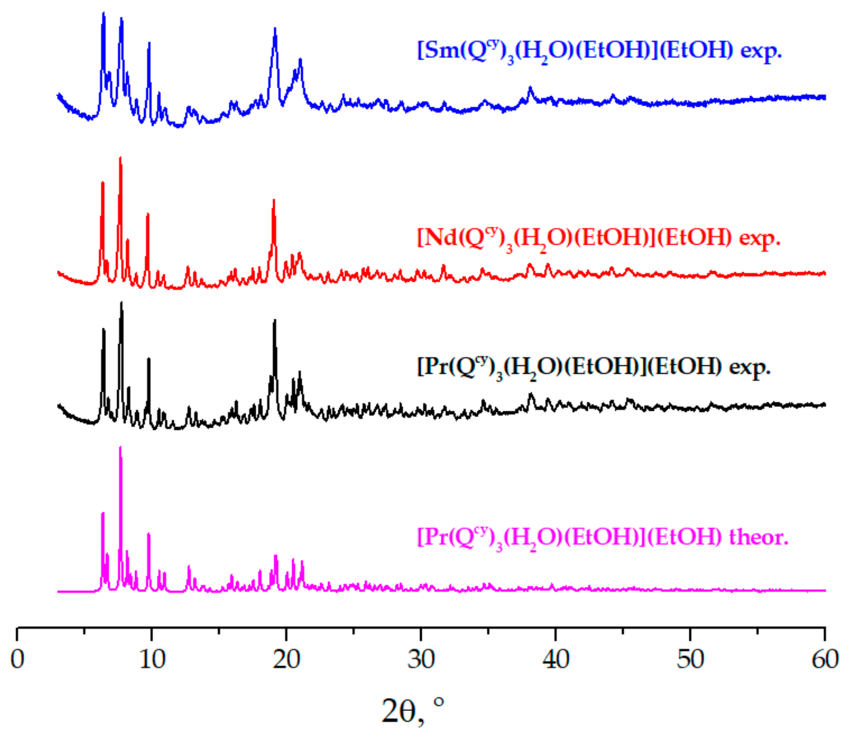

Figure S6. PXRD patterns of tris-complexes of Pr, Nd and Sm and simulated from single crystal data of Pr complex.
